# The role of lipids in neuromodulation for psychiatric disorders: A narrative review

**DOI:** 10.1038/s41398-026-03873-2

**Published:** 2026-02-08

**Authors:** D. M. Karaszewska, M. van Kesteren, I. Bergfeld, A. Lok, J. Assies, A. Dols, P. van den Munckhof, R. Schuurman, D. Denys, R. J. T. Mocking

**Affiliations:** 1 Department of Psychiatry, Amsterdam UMC, location Academic Medical Center, University of Amsterdam, Amsterdam, the Netherlands; 2 Department of Psychiatry, division Brain, UMC Utrecht, Utrecht, the Netherlands; 3 Department of Neurosurgery, Amsterdam UMC, location Academic Medical Center, University of Amsterdam, Amsterdam, the Netherlands

**Keywords:** Physiology, Depression, Schizophrenia, Biomarkers, Scientific community

## Abstract

Lipids are highly abundant in the brain and play key roles in membrane regulation, neurotransmission, neurogenesis, and inflammation. The same processes are involved in neuromodulation mechanisms. While neuromodulation therapies have shown promising outcomes for treatment-resistant psychiatric disorders, the factors determining individual variability in treatment response remain poorly understood. Furthermore, the potential impact of neurometabolic factors in predicting response has been largely overlooked. This narrative review aims to evaluate the role of lipids in psychiatric neuromodulation. Particularly glycerophospholipids, sphingolipids and polyunsaturated fatty acids (PUFAs) have been described as important mediators. Current evidence suggests a bidirectional relationship between lipids and neuromodulation therapies such as electroconvulsive therapy (ECT), and repetitive transcranial magnetic stimulation (rTMS). Neuromodulation effects are associated with lipid metabolism changes, including phospholipids, sphingolipids, and fatty acids. ECT is associated with an increase in lipid peroxidation and alterations of cholesterol and fatty acid levels, while rTMS is associated with normalization of sphingolipids and phospholipids levels. Solely one study investigated the relation between deep brain stimulation and lipids, showing an association with sphingolipid metabolism. To our knowledge, this is the first comprehensive review to consolidate findings on the relationship between lipids and neuromodulation. By mapping this emerging field, these findings might be a first step towards investigating whether lipids could be a potential biomarker for response prediction in the future. As most findings are preliminary, with variability across studies, further investigation is warranted and current findings should be interpreted in the context of their limitations.

## Introduction

The global burden of psychiatric disorders, measured in disability-adjusted life years (DALYs), increased from 80.8 million to 125.3 million between 1990 and 2019 [1]. Whilst existing treatments such as psychotherapy and medications are efficacious for many people, some need subsequent or additional therapies based on neuromodulation [2], including deep brain stimulation (DBS), electroconvulsive therapy (ECT) and repetitive transcranial magnetic stimulation (rTMS). Neuromodulation is an evidence based treatment for several psychiatric disorders such as treatment-resistant depression (TRD), increasingly being referred to as difficult to treat depression [3], or treatment-resistant obsessive-compulsive disorder (TR-OCD). The effect sizes are especially large considering that other treatments have been ineffective for these patients [4, 5].

Although, the exact neurobiological underpinning of neuromodulation are yet to be clarified, there is one factor that has been largely overlooked: neurometabolic factors such as lipids. Lipids make up more than half of the brain dry weight and are important determinants of the brain’s electrochemical properties. Lipids are the main component of all neural membranes and determine cell signaling, neural plasticity and inflammation processes [6]. Lipid alterations and disturbances in lipid composition have been associated with psychiatric disorders such as major depressive disorder (MDD), bipolar disorder (BD) and schizophrenia [7, 8].

Lipids were also longitudinally associated with treatment response of antidepressants suggesting causality [9]. A few pioneering studies successfully examined the relationship between neuromodulation and lipidome [10–13]. As neuromodulation therapies may exert their clinical effects through shared neurobiological mechanisms, which also underlie various psychiatric disorders, this overlap likely explains why different neuromodulation techniques demonstrate transdiagnostic efficacy across multiple psychiatric disorders. All in all, evidence suggests a bidirectional relationship between lipids and neuromodulation. However, so far, no overview of on the relationship between lipids and neuromodulation has been published.

In this literature review, we examine the bidirectional relationship between lipids and neuromodulation in patients with psychiatric disorders. As such, we provide a comprehensive overview of neuromodulation techniques in psychiatry and the major classes of brain lipids relevant to neural function. We limited our review to DBS, ECT and rTMS because these techniques are most widely used, evidence-based neuromodulation treatments in psychiatric practice and because the currently available lipidomic literature in psychiatric neuromodulation is concentrated around these methods. However, other neuroregulatory interventions may also be related to lipids and merit consideration for future research. Subsequently, we summarize the emerging evidence on the bidirectional relationship between lipids and neuromodulation in psychiatry, detailing how ECT, DBS, and rTMS affect lipid metabolism and how lipid profiles are, in turn, associated with neuromodulation. In the final section, we propose potential directions for future research and critically evaluate the limitations of the existing evidence.

## Methods

We conducted this narrative review by first summarizing the literature on neuromodulation and the metabolism of several classes of brain lipids. In the second part of our narrative review we included studies that focused on the combination and/or overlap between neuromodulation and lipids. Using Boolean operators, we combined search terms regarding neuromodulation (e.g., deep brain stimulation, transcranial magnetic stimulation, electric stimulation) and lipids (e.g., lipidomic, membrane lipids, phospholipase), and performed an explorative search in EMBASE, OVID and PsycINFO. Additional relevant literature was included through the snowballing method. We included studies that focused on the relationship between neuromodulation treatments (e.g., deep brain stimulation, transcranial magnetic stimulation, electric stimulation) in psychiatric disorders, and lipids. Studies that did not focus on the interaction between lipids and neuromodulation in psychiatric disorders were excluded.

## Results

Before providing the evidence on the interaction between lipids and neuromodulation therapies, it is crucial to first outline the principal neuromodulation treatments used in psychiatry (Section 3.1) and the fundamental roles of lipids in brain structure and function (Section 3.2). This narrative overview provides the necessary context for understanding how ECT, DBS, and rTMS may be associated with lipids, bidirectionally (Sections 3.3–3.5).

### Neuromodulation

In DBS neural activity is modulated invasively, whereas neural activity is modulated non-invasively in ECT and rTMS. All three techniques show long-term biological effects through affecting transcription factors, neurotransmitters, and neurotrophic factors, which influence together neuroplasticity, neurogenesis and neuroinflammation [14]. However, much remains to be studied to better understand these techniques, as it is currently unknown why some patients respond to neuromodulation therapies while others do not.

#### Deep brain stimulation

**Fig. 1:**
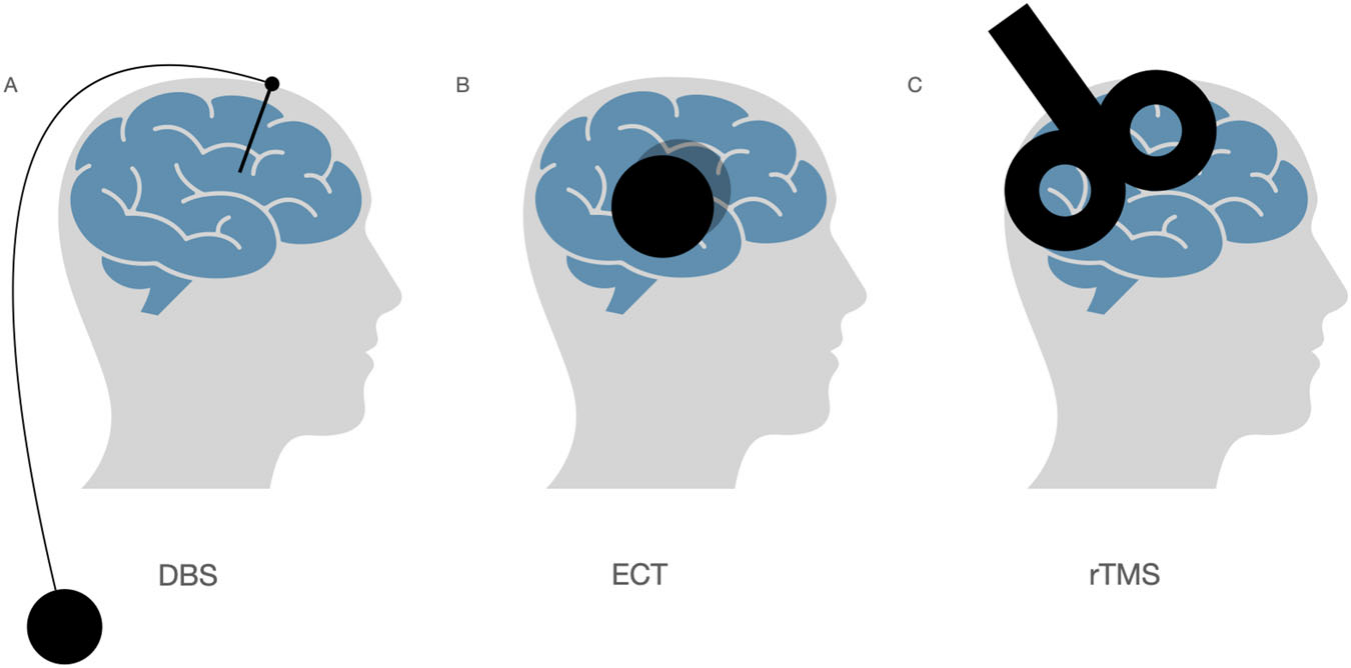
Different neuromodulation treatments. **A** DBS delivers electrical pulses to the brain through invasive electrodes. The pulses are generated at a high frequency. **B** During ECT, small electrical currents that are running between two electrodes are delivered to the brain, while the patient is under general anesthesia. **C** rTMS is a noninvasive brain stimulation technique that induces trains of short electromagnetic pulses on the scalp to reduce or increase cortical excitability. Pulses are generated by a constantly changing electromagnetic field.

DBS involves lifelong delivery of electrical pulses to targeted brain regions via intracerebral electrodes (Fig. 1A). These high-frequency pulses aim to restore abnormal neural circuits to a more physiological state [2], for instance by altering neurotransmitter release [15]. Some effects may become chronic through mechanisms like long-term depression or potentiation [16–19]. DBS shows response rates in TRD patients that range from 50% after a year until 65% after three years. Notably, DBS applied closer to white matter tracts was associated with better response in depression [20]. This is also shown in OCD, where the anterior limb of the internal capsula as a target site was shown superior [21]. In TR-OCD, response rates range from 50% to 60%, with about 75% of patients showing partial improvement in the first year, often sustained over time [4]. In Gilles de la Tourette syndrome, DBS leads to a 50% reduction in Yale Global Tic Severity Scale scores after one year [22].

#### Electroconvulsive therapy

During ECT small electric currents are delivered to the brain (Fig. 1B), under sedation or general anesthesia. These currents depolarize neurons resulting in a seizure that is necessary for the therapeutic effect of ECT. It is thought that the seizure initiates the resetting of altered non-functional brain connectivity leading to changes in neurotransmitter release [17, 23]. ECT is utilized to treat MDD, schizophrenia and catatonia [24]. Response rates to ECT in MDD are approximately 60%, whereas in OCD the response rates are 79%. However, the evidence on OCD is limited to case studies, and relapse occurred in 35–55% of the cases [25, 26].

#### Transcranial magnetic stimulation

rTMS induces trains of short electromagnetic pulses on the scalp to reduce or increase cortical excitability, at low (<1 Hz) and high frequencies (>5 Hz) respectively [2]. These pulses are generated by a constantly changing electromagnetic field (Fig. 1C). The effect of rTMS is believed to be less precise than of DBS [2]. Response rates to rTMS range from 40 to 60% in MDD [27–29] and from 35 to 45% in obsessive-compulsive disorder (OCD) and Tourette Syndrome (TS) [30–32].

### Lipids

**Fig. 2:**
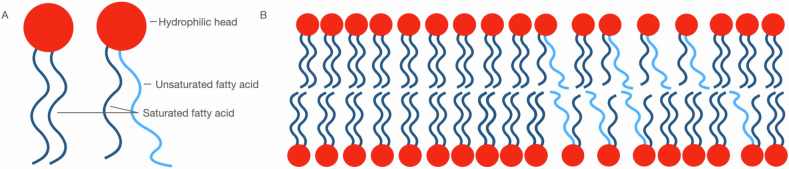
Phospholipids are main components of neural membranes. **A** Phospholipids contain a hydrophilic head and two hydrophobic tails that is either saturated or unsaturated. **B** The degree of FAs saturation determines the fluidity of the membrane.

The effects on the following lipids were examined: phospholipids, fatty acids (FAs), glycerolipids, glycerophospholipids, sphingolipids and sterol lipids (cholesterol) [10]. Phospholipids are the main components of neural membranes (Fig. 2A). Furthermore, FAs determine the saturation and therewith fluidity of membranes (Fig. 2B) [33]. Decreased fluidity is associated with impaired signal transduction [34]. Moreover, nervonic acid (NA, fatty acid) is one of the main components of white matter [35]. NA is involved in the synthesis of sphingomyelin (SM), that is a major component of myelin, by binding to sphingosine via amide bonds [35]. NA not only plays a role in the formation of myelin, but also in maintaining its normal functioning. Also, lipids can mediate signal transduction by acting as ligands, for example after being cleaved from the membrane by enzymes called phospholipases [6]. Disturbances in neural signaling, therewith potentially in lipid levels could play an important role in psychiatric disorders. Aspects of lipid metabolism possibly involved in both psychiatric disorders and neuromodulation are described below [36].

#### Fatty acids

Polyunsaturated fatty acids (PUFAs) constitute 35% of the brain dry weight [37]. Particularly long-chain n-3 and n-6 PUFAs are essential for the brain and must be obtained from the diet [6, 38]. Docosahexaenoic acid (DHA) and eicosapentaenoic acid (EPA) are important n-3 PUFAs, while arachidonic acid (AA) is an essential n-6 PUFA [39]. DHA is the most prevalent PUFA in neural membranes. It has anti-inflammatory properties [40], which can be interesting for psychiatric therapies as inflammation is often increased in psychiatric disorders such as MDD, BD and schizophrenia [41, 42]. DHA has an important role in precise electrochemical cell signaling (through quantum mechanics) due to its properties, which may explain its enrichment in the brain and retina [43]. Moreover, EPA is also anti-inflammatory [44, 45], whereas AA is rather pro-inflammatory [46–48]. Both FAs are known for their modulatory role on the duration and intensity of immune responses [49]. In short, PUFAs are involved in membrane dynamics, fluidity, neurogenesis and neural plasticity [50–55], neural signaling (neurotransmission, even at genetic level [56, 57]), maintaining brain activity [58] and anti-inflammatory processes [7, 59]. They can protect neurons from excessive firing by modulating neuronal excitability.

#### Lipid peroxidation

Lipid peroxidation, a process by which oxidants attack lipids, can affect all classes of lipids, but especially PUFAs [60]. The process is therefore described in this chapter. Oxidants, such as free radicals, arise under high levels of oxidative stress. Oxidation of PUFAs can impair neuronal membranes, where PUFAs mainly reside. If oxidative stress levels are too high, the capacity of a neuron’s antioxidant defense system is too low resulting in neuronal cell damage [60]. This process of lipid peroxidation has been associated with several psychiatric disorders, such as schizophrenia and bipolar disorder [61].

#### Glycerophospholipids

Neural membranes consist of 75% glycerophospholipids [62, 63]. Glycerophospholipids exert their functions largely through membrane dynamics, for instance influencing neuronal excitability [6] and neurotransmitter communication through vesicles fusion stimulation, which might be impaired in psychiatric disorders [64]. In addition, glycerophospholipids regulate membrane fluidity through recruiting PUFAs [38]. The composition of proteins in the membrane influences neurotransmission, which is the target in many psychiatric drugs [65].

#### Sphingolipids

Sphingolipids consist of ceramide linked to either saccharides or phosphocholine, forming glycosphingolipids (GSLs) or sphingomyelin (SM), respectively. Gangliosides are a specific type of GSLs containing sialic acid. They account for 10-12% of the lipids in neural membranes, and are to be found in both grey matter, neurons and white matter, and are important determinants of white matter integrity [66, 67]. Sphingolipids are best known for their role in the synthesis and maintenance of myelin, anti-inflammatory processes and neural plasticity [11, 67, 68]. Normal myelin genesis may be interrupted in MDD and schizophrenia [69, 70].

#### Cholesterol

Cholesterol is a sterol that is synthesized in neurons and astrocytes. This sterol is mainly found in membranes of myelin, but also in membranes of astrocytes and neurons[71]. In the brain, cholesterol plays a role in the stability of membranes and in the formation of synapses and dendrites [71]. Cholesterol is important for membrane stability as it organizes the ordering of phospholipids affecting both membrane curvature and fluidity [72]. Cholesterol is therefore also involved in vesicle exocytosis [73]. Furthermore, cholesterol functions as a precursor of estradiol which has been linked repeatedely to the formation of synapses via activity-dependent mechanisms [74]. These functions all indicate that depletion of cholesterol results in impaired neurotransmission. Further studies are however required to understand the exact functioning of cholesterol [71].

In summary, lipids fulfill a broad spectrum of electrochemical roles within the brain. Due to the significant overlap between these functions and the mechanisms of neuromodulation, the subsequent section will focus specifically on this intersection.

### Lipids and neuromodulation

Pioneering research has explored the bidirectional relationship between lipids and neuromodulation, investigating both how neuromodulatory processes affect lipid dynamics and how lipids, in turn, influence neuromodulatory signaling. Notably, several mechanisms underlying neuromodulation exhibit considerable convergence with the electrochemical properties of lipids.

#### Effects of neuromodulation on lipids

##### Effects of ECT on lipids

*Animal ECT studies:* During the late 80’s, It was found that electroconvulsive seizures (ECS) increased the amount of free FAs in rats’ brains [75]. Levels of AA increased in mice brains [76] and cholesterol concentrations, increased in plasma but diminished in red blood cells [77]. These studies were the first to examine lipids following neuromodulation. Also, phosphatidylinositol (a glycerolphospholipid) was increased in rats after ECS [78].

Furthermore, studies performed on rats showed that ECS increased lipid peroxidation in the frontal cortex, while others found similar results in the PFC and hippocampus [79]. Moreover, a study on ECS performed in rats showed an increase in lipid peroxidation in the frontal cortex, when comparing to the control group, but not in other brain areas, such as hippocampus, cerebellum and pons/medulla [79].

*Animal research and ECT: mechanisms of action*: Animal studies can provide insights into neurobiology of psychiatric disorders and the mechanism of action of neuromodulation therapies. However, the neuroanatomical differences between animals and humans need to be taken into account, while interpreting the results.

A hypothesis for an increase in FAs levels in rats following ECT is activation of membrane phospholipase A through ECS splitting FAs from the membrane retaining their properties [80, 81]. Also, ECT plausibly modifies neuronal cell signaling, either by means of inducing a seizure or through simulation of the electric field induced in the brain and the release of second messengers [78, 82]. For example, researchers found regionally specific changes in phospholipase C levels, after repeated ECT, indicating effects in cell transduction, therewith possibly initiating lipid levels changes [83]. Finally an increase in lipid peroxidation was seen in rats after ECT, pointing towards oxidative lipid damage. However, when rats received a combination therapy of ECT together with either antidepressants or ketamine, a reduction in lipid peroxidation was seen, showing possible neuroprotective effects of antidepressants or ketamine [84].

Additionally, the effects of ECT on lipids were studied on a genetic level. In rats, electroconvulsive shocks increased the transcription of genes involved in the AA cascade in two studies [85, 86]. These results might provide one perspective explaining how ECT can exert long-term effects, showing an influence on inflammatory relevant AA metabolism.

*Human ECT studies:* One recent multicenter study including 45 MDD patients found longer fatty acids to be higher in late responders compared to non-responders. Furthermore NA levels were higher in late responders, compared to early, and non-responders [87].

Also, three human studies found increased levels of total cholesterol after ECT in MDD, bipolar mood disorder and schizophrenia, leading to serum lipid profile changes. However, regarding HDL and LDL levels the results are contradictory [88–90].

Furthermore, a clinical study on lipidomics performed in 16 MDD patients undergoing ECT, found 69 significantly altered lipid metabolites after ECT. Free FAs in particular were reduced after treatment [91]. However, contradictory results do not lead to one conclusion. For instance, one study on TRD patients (n = 38) showed no alterations in serum lipids after a single ECT session, nor during a whole course of ECT [92]. Table 1 provides an overview of animal and human studies on ECT and lipids.

**Table 1:** Overview of results found on the association between ECT and lipids.

	Animal studies on ECT and lipids	Human studies on ECT and lipids
Lipids		No alterations in serum lipids after a single/whole course of ECT were found in MDD [92]
Fatty acids	Electroconvulsive shocks increased free fatty acids (FAs) in rats’ brains [75] Levels of arachidonic acid (AA) increased in mice brains [76]	A higher chain length index of fatty acids was found in late responders compared to non-responders and NA levels were also higher in late responders compared to early- and non-responders in MDD [87] Free fatty acids (FAs) were specifically reduced in MDD [91]
Cholesterol	Cholesterol levels increased in plasma but diminished in red blood cells [77]	Cholesterol levels increased in MDD, bipolar mood disorder and schizophrenia [88–90]
Phospholipids	Phosphatidylinositol increased in rats [78]	
Lipid peroxidation	An increase in lipid peroxidation was found in rats, in the frontal cortex in particular [79]	

##### Effects of rTMS on lipids

*Animal rTMS studies*: A study with rats exposed to chronic unpredictable stress (CUS) showed that rTMS normalized sphingolipids levels, including ceramides, glucosylceramides, ceramide phosphate, and sphingomyelin. Also, glycerophospholipid levels were normalized. More specifically, phosphatidylethanolamine, phosphatidylinositol and lysophosphatidylcholine decreased, while phosphatidic acid increased. However, glycerolipid levels were only normalized in the hippocampus, while less so in the prefrontal cortex (PFC). Fatty acid levels were not at all [13].

A similar experiment was performed using mice exposed to demyelination. In these mice, sphingolipid concentrations were normalized. Likewise, glycerophospholipid levels were normalized. The effects were region-specific, as the effects on lipids were particularly observable in the hippocampus while less so in the striatum and PFC. Yet, glycerolipids and glycerosyldiaglycerols levels were not affected by rTMS. (8)

In a study with healthy rats receiving five days of rTMS, some phospholipids decreased in the PFC, while others decreased. Especially the ones with longer chains showed a decreasing effect. However, most sphingolipids increased in the PFC after rTMS. These effects in the PFC were opposite to the effects in the striatum. No effects of rTMS were found in the hippocampus. Furthermore, no large differences were found between the hemispheres. In general, it was found that the dose of rTMS was linearly related to the effect of the lipids. (87)

*Animal research and rTMS: mechanisms of action*: Both rTMS and lipid levels have been associated with oxidative stress. Combining these lines of research, it was found that oxidative stress increased lipid peroxidation products that were subsequently reversed by TMS, in rats [93, 94]. These results suggest that TMS reduces oxidative stress by means of antioxidant actions, which might improve the therapeutic effect [95]. Besides antioxidant processes, research shows neuroprotective effects in rats after rTMS, through acting on stress hormones, dopamine, serotonin, brain derived neurotrophic factor (BDNF) expression, neuroinflammation, and hippocampal cell proliferation [93, 96].

*Human rTMS studies*: The above animal studies have also been investigated, however to a lesser extent, in humans. One study, used human blood samples and found a decreasing effect of cholesterol and triglycerides after rTMS in aging adults [97]. Similar results were found in a retrospective observational study in 34 TRD patients, with decreasing levels of total HDL and LDL cholesterol levels [98]. Another study, investigating lipids in TRD patients, showed that niacinamide levels in the cerebrospinal fluid (CSF) were increased after rTMS. Niacinamide is related to lipid level alterations [99]. In a study including 30 patients with bipolar disorder, several medium- and long-chain fatty acids levels increased after 2 weeks of treatment with rTMS combined with quetiapine and mood stabilizer intervention [100]. An overview of both animal and human studies is shown in Table 2.

**Table 2:** Overview of results found on the association between rTMS and lipids.

	Animal studies on rTMS and lipids	Human studies on rTMS and lipids
Lipids		Niacinamide levels in CSF, related to lipid level alterations, increased after rTMS in TRD patients (93)
Fatty acids	Fatty acids levels were not normalized in rats exposed to CUS (11)	Levels of total medium- and long-chain fatty acids increased after two weeks of treatment with rTMS, combined with quetiapine and a mood stabilizer [100]
Phospholipids	Phospholipids were affected by rTMS in healthy rats (87) Glycerophospholipids were normalized in a rat model of CUS (11) and in mice exposed to demyelination (8).	
Glycerolipids	Glycerolipids were partially affected by rTMS in rats exposed to CUS (11) Glycerolipids were not normalized in mice exposed to demyelination (8)	Triglycerides in blood were decreased in aging adults (92)
Glycerosyl-diaglycerols	Glycerosyldiaglycerols levels were not affected by rTMS in mice exposed to demyelination (8)	
Sphingolipids	Sphingolipids were normalized in rats exposed to CUS (11) and in mice exposed to demyelination (8). Sphingolipids were affected by rTMS in healthy rats (87)	
Cholesterol		Cholesterol in blood was decreased in aging adults. (92) Also, a decrease in total, HDL and LDL cholesterol levels was found in patients with TRD [98]
Lipid peroxidation	Lipid peroxidation products were decreased in rats with increased oxidative stress (88, 89)	

##### Effects of DBS on lipids

One study investigated the effect of DBS on lipid metabolism. DBS increased sphingolipid concentrations, such as HexCer and LacCer in the hippocampus of rats. Both lipids are known to be involved in membrane formation. No effect of DBS on glycophospholipids was observed. There was no difference in DHA concentration before and after DBS [11].

#### Summary of effects of neuromodulation on lipids

Taken together, neuromodulation therapies and lipid dynamics appear to be interconnected through multiple biological pathways. These include modulation of neuronal signalling, neurotransmission, and intracellular signal transduction, as well as alterations in systemic lipid profiles and concentrations of lipid metabolites, fatty acids, and sphingolipids. Additionally, evidence from studies involving ECT and DBS indicates that lipid peroxidation is modulated following treatment, suggestive of oxidative stress involvement. In parallel, transcranial magnetic stimulation (TMS) has been associated with reductions in oxidative stress, potentially mediated by upregulation of endogenous antioxidant mechanisms. Nevertheless, the mechanistic relationship between neuromodulation, oxidative stress, and psychiatric pathology remains insufficiently elucidated.

### Effects of lipids on neuromodulation

Given the close interplay between neuroinflammatory pathways and lipid signaling, particularly through lipid mediators such as sphingolipids and PUFAs, these immunomodulatory effects of neuromodulation suggest a potential mechanistic link to lipid metabolism. Lipids not only modulate neuroinflammatory responses but also serve as critical components in cell signaling, membrane dynamics, and oxidative stress, all of which are influenced by neuromodulatory interventions.

Limited research has been conducted on how lipids influence neuromodulation. In an explorative human study, it was observed that classes of oxidized phosphatidylcholines (OxPCs) were higher in MDD remitters than in non-remitters to rTMS. No effects of oxidized FAs were found [101]. In another study, PUFA chain length was positively correlated with ECT effectiveness in MDD patients. It was hypothesized that long-chain PUFAs make neural membranes more sensitive to electrical stimuli. Besides, NA concentrations were higher in late-responders compared to early- and non-responders. This may be explained by myelin, being formed by NA, on which electrical stimulation is the most effective [102]. Moreover, a positive correlation between the level of sphingolipids at baseline and antidepressant efficacy was observed in depressive patients during ECT, albeit at trend level significance [103].

## Discussion

This review explores the bidirectional relationship between lipids and neuromodulation therapies in psychiatry. Neuromodulation therapies are effective transdiagnostically, suggesting overlap in underlying pathophysiological mechanisms. Neuromodulation and lipid metabolism seem bidirectionally related. Based on our findings, neuromodulation exerts diverse effects on lipid metabolism. Across studies, ECT is most consistently associated with changes in arachidonic acid, cholesterol, and markers of lipid peroxidation, reflecting both oxidative and membrane-remodelling processes. rTMS studies report normalization of lipid peroxidation products and modulation of phospholipids and sphingolipids. Finally, given the absence of replication or human data, conclusions on the association between DBS and lipids should be regarded as highly preliminary and hypothesis-generating. Overall, these modality-specific findings imply dynamic relationships between neuromodulation and lipids.

The findings reviewed here suggest that multiple biological mechanisms may underlie the interaction between lipid metabolism and the therapeutic effects of neuromodulation. One key pathway involves oxidative stress regulation, as evidenced by alterations in lipid peroxidation following ECT and DBS. In parallel, TMS has been associated with antioxidant effects, potentially contributing to its therapeutic efficacy. A second mechanism may involve myelination processes. Myelin, predominantly composed of sphingolipids, is essential for rapid signal conduction, and its structural and functional integrity is regulated in part by noradrenaline (NA) [104]. Neuromodulation may exert its effects by modulating these lipid-based processes, thereby enhancing neural conductivity and circuit efficiency.

TMS, ECT and DBS have been reported to improve the neuroinflammatory status associated with various psychiatric disorders. Neuroinflammation is increasingly recognized as a core feature in the pathophysiology of MDD, bipolar disorder, and schizophrenia, with elevated levels of pro-inflammatory cytokines such as tumor necrosis factor-alpha (TNF-α), interleukin-6 (IL-6), and interleukin-8 (IL-8) commonly observed in affected individuals. Several studies have documented that neuromodulatory interventions can attenuate these markers of immune dysregulation. Both ECT and rTMS have demonstrated anti-inflammatory effects, for instance by significant decreases in serum IL-6 and IL-8 levels following ECT [14]. Although less extensively explored, DBS has also been linked to favourable immunomodulatory outcomes. For example, studies in patients undergoing DBS for TR-OCD have reported changes in TNF-α and IL-6 levels in plasma serum [105]. These findings might be interesting in the context of lipids, particularly due to a strong relationship between lipids and inflammation [106].

The electrophysiological mechanisms by which neuromodulation alters lipid metabolism remain poorly understood. DBS, ECT and rTMS modulate neuronal excitability and membrane potential, processes intimately linked to lipid signalling [107, 108].

Many ion channels and transporters depend on phosphatidylinositol-4,5-bisphosphate (PIP₂) as a cofactor; rapid hydrolysis of PIP₂ by phospholipase C alters channel gating within milliseconds, coupling excitability changes to acute shifts in membrane lipid composition. Over longer timescales, sustained neuromodulation may engage structural and systemic lipid pathways [109]. In animal models, neuronal activity promotes oligodendrogenesis and myelin lipid turnover, while activity-dependent activation of sterol regulatory element-binding proteins (SREBPs) modulates cholesterol and fatty acid synthesis in dendrites and synapses [110–112]. At the systemic level, neuromodulation may influence lipid metabolism through neuroendocrine axes such as the hypothalamic–pituitary–thyroid pathway. Clinically, subthalamic DBS in Parkinson’s disease has been associated with a mean weight gain of ~6 kg after one year, potentially reflecting alterations in hormonal balance, energy expenditure, and lipid oxidation [113]. Neuromodulation thus likely operates not only via circuit-level electrical effects but also through lipid-dependent molecular and metabolic cascades. Standardized studies are needed to clarify these electrophysiological–lipid interactions and their causal significance.

Despite these promising insights, current evidence remains limited and heterogeneous across studies, limiting the ability to draw conclusions for clinical practice. The effect of lipids on neuromodulation has only been studied in humans, suggesting characteristics like PUFA chain length may predict ECT treatment response in MDD. Despite limited data, findings support further investigation into lipid–neuromodulation interactions. Nonetheless, emerging evidence suggests that PUFAs may enhance neuronal membrane excitability by increasing membrane fluidity and sensitivity to electrical stimulation. Similarly, high concentrations of NA appear critical for effective action potential propagation, likely through their role in promoting myelin synthesis and stability. Indeed, structural changes in myelin have been proposed as mediators of therapeutic response to ECT, rTMS, and DBS in MDD. Supporting this, alterations in white matter connectivity, as captured by brain connectome analyses, have been correlated with clinical outcomes [114].

### Implications for future research

This literature review highlights relevant insights for future research. First, specific lipids could be of particular interest in the context of response prediction research to neuromodulation therapies. For clinical practice, biomarker research is relevant in order to improve patient selection, and thereby response rates. Second, lipids, especially n-3 PUFAs, show promise as add-on therapies for psychiatric disorders like MDD, ADHD, and borderline personality disorder by restoring lipid metabolism, membrane fluidity, and neurotransmission, ultimately increasing the accessibility and efficacy of therapeutic agents [115, 116]. This rationale may extend to neuromodulatory interventions, as patients with psychiatric disorders often exhibit disturbances in lipid composition and turnover, potentially influencing neuroplasticity and circuit responsiveness to stimulation. Moreover, the relationship between different stimulus parameters and lipids might be of particular interest for future research.

Additionally, gut microbiota influence systemic and brain lipid profiles and inflammation, both relevant to treatment response and psychiatric symptomatology. As such, future research could explore the synergistic potential of microbiota-targeted interventions, such as prebiotics, probiotics, or microbiome-informed dietary strategies, in enhancing the bioavailability and neuromodulatory impact of PUFAs. Overall, lipids, within host–microbiome dynamics, emerge as promising tools for integrative mechanism-based augmentation of neuromodulation in precision psychiatry [117].

### Limitations and strengths

This literature review is the first to summarize emerging evidence on the relationship between brain lipids and neuromodulation, aiming to inspire future research. Nevertheless, due to the pioneering stage of the research field, we included studies with heterogeneous populations, varying animal models, and differing neuromodulation protocols. Many studies had preliminary designs and lacked correction for multiple testing, warranting cautious interpretation. Most research was conducted in animals, complicating translation to humans, which is especially challenging since lipids are extracted from brains in rodents, while in humans these lipids are extracted from peripheral blood. Human studies, based on small sample sizes, further limit clinical interpretation. Larger sample sizes are needed to clarify the clinical significance of the findings. Finally, the majority of included human studies are observational, including relatively small sample sizes, therefore the reported associations should be interpreted as correlations.

## Conclusion

Current evidence suggests a bidirectional association between lipids and neuromodulation. Glycerophospholipids, sphingolipids, and PUFAs may be important mediators for the effects of neuromodulation. However, due to the observational or exploratory designs in the included studies, the results should be interpreted with caution in the context of causality. This review highlights the relevance for more research, including investigation into the potential of lipids as predictors of treatment response.
